# Pathogenomic Insights into *Xanthomonas oryzae* pv. *oryzae*’s Resistome, Virulome, and Diversity for Improved Rice Blight Management

**DOI:** 10.3390/life14121690

**Published:** 2024-12-20

**Authors:** Peter Adeolu Adedibu, Oksana Son, Liudmila Tekutyeva, Larissa Balabanova

**Affiliations:** 1 Institute of Biotechnology, Bioengineering and Food Systems, Advanced Engineering School, Far Eastern Federal University, 10 Ajax Bay, Russky Island, 690922 Vladivostok, Russia; 2 ARNIKA, Territory of PDA Nadezhdinskaya, Centralnaya St. 42, Volno-Nadezhdinskoye, Primorsky Krai, 692481 Vladivostok, Russia; 3 G.B. Elyakov Pacific Institute of Bioorganic Chemistry, Far Eastern Branch, Russian Academy of Sciences, Prospect 100-Letya Vladivostoka 152, 690022 Vladivostok, Russia

**Keywords:** bacterial rice blight, *Xanthomonas oryzae*, *Oryza sativa*, plant pathogen, resistome, virulome, genetic diversity

## Abstract

*Oryza sativa* (rice) is a major staple food targeted for increased production to achieve food security. However, increased production is threatened by several biotic and abiotic factors, of which bacterial blight disease caused by *Xanthomonas oryzae* pathovar *oryzae* is severe. Developing effective control strategies requires an up-to-date understanding of its pathogenomics. This study analyzes the genomes of 30 *X. oryzae* strains collected from rice-producing regions across five continents to identify genetic elements critical for its pathogenicity and adaptability and for an intraspecific diversity assessment using advanced genomics and bioinformatics tools. Resistome analysis revealed 28 distinct types of antibiotic resistance genes (ARGs), both innate and acquired, indicating a growing threat from multidrug-resistant *X. oryzae* strains. Sixteen virulent genes, including type III and VI secretion systems, motility genes, and effector proteins, were identified. A unique ‘*MexCD-OprJ*’ multidrug efflux system was detected in the Tanzanian strains, conferring resistance to multiple antibiotic classes. To curb further ARG emergence, there is a need to regulate the use of antibiotics for *X. oryzae* control and adopt resistant rice varieties. Transposable elements were also discovered to contribute to *X. oryzae* pathogenicity, facilitating the horizontal transfer of virulence genes. Pangenome analysis revealed intraspecific variation among the population, with 112 unique CDS having diverse functional roles. Strains registered in the Philippines had the most unique genes. Phylogenetic analysis confirmed the divergent evolution of *X. oryzae*. This study’s results will aid in identifying more effective management strategies and biocontrol alternatives for sustainable rice production.

## 1. Introduction

*Oryza sativa* (rice) is a major staple food in many world regions, globally cultivated and feeding approximately half of the world population [1]. It is very popular and crucial for food security in developing and advanced nations, accounting for a sizeable amount of the daily caloric intake [2]. As the world strives to avoid the looming food crisis from the increasing global population, rice is one of the staple foods of interest targeted for increased production to achieve food security [3]. However, several biotic and abiotic stress factors threaten increased rice production, limiting its yield globally. Among the most severe biotic threats is bacterial blight caused by the *Xanthomonas oryzae* pathovar *oryzae*.

*Xanthomonas* spp. are recognized as among the top economically important bacteria plant pathogens, infecting not less than 268 dicotyledonous and 124 monocotyledonous plants [4,5]. *X. oryzae* is an important rice pathogen and a significant barrier to enhanced rice production globally, causing rice disease bacterial blight [6,7] prevalent in tropical and temperate climates [4]. The pathogen gains entry into rice leaves via wounds, natural openings, and leaf hydathodes. It spreads through the xylem, causing extended, opaque necrotic lesions that range from white to grey [8]. *X. oryzae* causes terrible yield loss and subpar grain quality, increasing the cost of production in rice-growing areas of the world [6]. The pathogen is receiving increasing scientific attention due to its devastating and yield-limiting impact on rice plantations, so more focus has been placed on understanding its virulence mechanisms and interaction with rice systems [9].

Previous studies have established the occurrence of several virulence factors in *X. oryzae* strains, such as extracellular enzymes, toxins, the type III secretion system and its effectors, adhesion, lipopolysaccharide (LPS), extracellular polysaccharides, biofilm and cell motility, and others, which are involved in its pathogenic process in the host rice tissue [10,11,12]. The pathogen exhibits notable genome flexibility with multiple virulence potential, making it adaptable to host plant defenses and complicating control efforts [13]. A recent study on the *X. oryzae* population in the Philippines reported new virulence patterns in adaptation to the resistance gene ‘*Xa4*’ in the improved IR20 rice variety introduced in the region [14]. In Zhejiang Province of China, three new strains emerged with greater virulence than the previous epidemic strain for which resistance rice varieties were already introduced [15]. Likewise, in Tanzania, new virulent strains of *X. oryzae* were identified, having TAL effectors typical to Asian strains, with a devastating impact on rice plantations in the region [16]. These emphasize the importance of constantly monitoring its virulence dynamics and emerging strains to safeguard rice crops against potential outbreaks.

The role of T3SS and transcription activator-like effectors (TALEs) in *X. oryzae*’s interaction with host plants has been proven through integrated genomic and transcriptomic approaches [17]. Through various mechanisms, these effector proteins ensure host–plant infection [10,12].

Genomic and bioinformatics advancements in recent years have made it possible to study *X. oryzae* and other plant pathogens in extensive detail and should be further explored [18,19]; these tools have revealed important genetic elements contributing to pathogenicity, resistance to antibiotics, and adaptation, including the mobile gene elements (MGE) and transposons, which also influence these traits [12]

Kaur et al. [20] highlighted the significant role of MGEs in driving genomic rearrangements and enhancing virulence in *X. oryzae* pathovars, further contributing to the emergence of novel pathotypes by facilitating genomic flux and acquiring new gene cassettes, which enhance pathogenicity. In a recent study, Fernandes et al. [21] identified many insertion sequences (ISs) and transposons within *X. oryzae* genomes, contributing to genetic variability and adaptability in response to host defenses. Integrons have also been reported to contribute to antibiotic resistance (AR) in *X. oryzae* [22]. The study established the role of class 1 integrons and the *aadA1* gene in conferring resistance to streptomycin and spectinomycin in the resistant isolates analyzed [22].

These MGEs facilitate the horizontal transfer of passenger genes such as virulence and AR genes in phytopathogens [23], fostering intra-specific evolution and adaptability to host environment demands [24,25]. Previous studies have established significant variation and divergent evolution among its population [26]. The comprehensive analysis of these genetic elements, their functional roles, and their impacts on the evolutionary dynamics of the pathogen population is indispensable in modern pathology.

The surge of AR in phytopathogens needs more attention than it has currently, especially with the increasing demand for antibiotics in crop protection. The use of antibiotics in crop protection is significantly underreported [27]. The Wellcome Trust estimated the annual use of antibiotics in agriculture to be approximately 240,000 tons, including the control of phytopathogens [28]. In certain instances, the use of antibiotics in crop protection has been estimated to be 700 times that used in human medicine [29,30]. *X. oryzae’s* resistome exhibits remarkable resistance to a wide range of antibiotic drugs and host-derived antimicrobial peptides, ensuring its survival in the host plant [31]. Very few reports exist on the AR genes profile of this pathogen [31,32], as its resistance mechanism grossly remains unexplored. This study closely examines the genetic elements that foster multidrug resistance.

This study provides a comprehensive analysis of the pathogenomics of the rice blight causative bacterium *X. oryzae*, analyzing virulent strains from rice-producing countries across continents. We aim to identify the genetic elements key to its pathogenicity and adaptability and assess its intraspecific phylogenetic profile using advanced genome analyses and bioinformatics tools. A thorough analysis and in-depth understanding of its virulence and evolutionary dynamics will help identify more effective strategies for *X. oryzae* management. The findings of this study will have a substantial impact on sustainable disease control and provide breeders with essential information for the development of resistant rice varieties and agrochemicals to curb the pathogen towards sustainable rice production.

## 2. Materials and Methods

### 2.1. Data Collection

**Figure 1:**
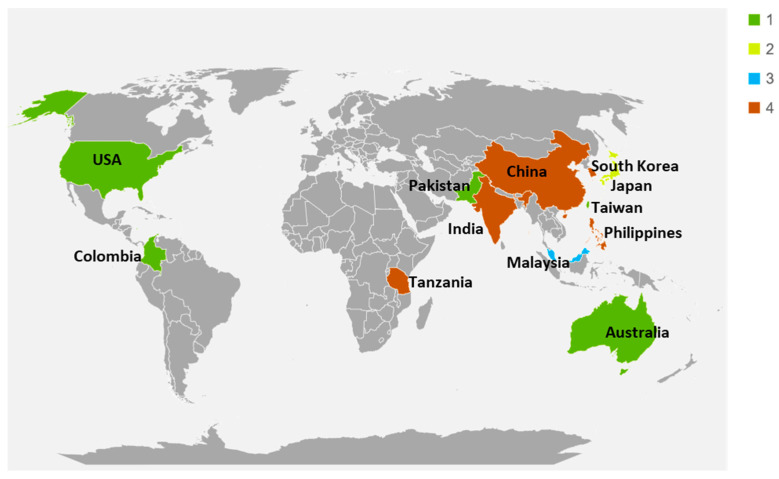
The geographical spread of *X. oryzae* strains was assessed in this study. Colors indicate the sample size.

This study utilizes full genomic sequences of 30 strains of *X. oryzae*, isolated from rice blight-infected *Oryza sativa* leaves from various locations across 12 countries and 5 continents, to conduct comprehensive functional genomic analyses of the pathogen. The samples were sourced from registered bioprojects in the public repository (https://www.ncbi.nlm.nih.gov/, accessed on 10 September 2024). The details of the analyzed samples, including the assembly information, GenBank accession numbers, geographical source, sequencing platform, and submitter information, are presented in Supplementary Table S1. Figure 1 gives a concise overview of the geographical spread of the strains assessed in this study.

### 2.2. Data Preprocessing

The WGS data were processed using the Bv-brc genome assembly pipeline (v3.6.9) [33], which employs Unicycler assembler (v0.4.8) [34] and Pilon (v1.23) [35] to improve assembly results and correct misassemblies. Assembly was carried out with a minimum contig length of 300 bp and a minimum contig coverage of 5.0. Assembly quality was evaluated by QUAST (v5.0.2) [36].

### 2.3. Genome Annotation and Subsystem Classification

All 30 *X. oryzae* genomes were annotated using Prokka v2.1.1 [37] and the genome annotation service (https://www.bv-brc.org/app/Annotation, accessed on 15 September 2024) on the Bacterial and Viral Bioinformatics Resource Center (BV-BRC) v3.37.14 server [38], which provides the annotation of genomic features using the Rapid Annotation function of the Subsystems Technology toolkit (RASTtk) [39,40] to predict genes, gene functions, and other features.

### 2.4. Antibiotic Resistance and Virulence Factor Genes Prediction

The comprehensive genome analysis service, available on BV-BRC v3.37.14 (https://www.bv-brc.org/app/ComprehensiveGenomeAnalysis, accessed on 15 September 2024), was used for antibiotic resistance genes prediction. The annotated genomes were uploaded and queried against the Pathosystems Resource Integration Center (PATRIC) ARG database [41,42] using hidden Markov models [43] to identify the ARGs. We further collected data on ARG types, gene products, and functional mechanisms to understand their prevalence among *X. oryzae* strains.

To identify Virulence Factor Genes (VFGs) in *X. oryzae* genomes, annotated genomes of the strains were screened for sequence similarity (with options: minid 70, mincov 70) between the VFGs and the known VFGs in the Virulence Factors Database for bacterial pathogens (VFDB) [44]. The prediction was made using Abricate (v 1.0.1) [45] hosted on the Galaxy platform (https://usegalaxy.org, accessed on 17 September 2024) [46]. Predicted virulence factors were categorized based on their roles.

### 2.5. Identification and Characterization of Mobile Genetic Elements

Annotated genomes of the strains were uploaded to two databases, ISfinder [47] and Prokaryotic Transposable Element Database and Web Portal for Transposon Analysis (Tncentral) [48], known for the detection of insertion sequences (IS) and transposons in bacteria genomes. Transposable elements were identified and annotated based on sequence similarity and a comparison with database-curated information. Initial predictions were filtered using an E-value cutoff of 0.00 to ensure the accuracy of the identified elements. Data from both platforms were cross-referenced and further analyzed to determine their distribution and influence on host strains.

### 2.6. Pangenome Analysis and Orthologous Group Identification

Pangenome analysis was conducted to unravel the genomic diversity and identify shared and unique gene contents among the 30 *X. oryzae* strains against the NCBI reference strain GX01 (GenBank accession number CP043403.1). NCBI BLAST 2.15.0 [49] was used to perform an all-against-all nucleotide BLAST comparison (BLASTn) among the strains using default parameters. Similar genes were predicted from the annotated genomes and clustered into orthologous groups to construct the pangenome using BRIG software (v1.0) [50]. A pangenome graph was built to depict the strains’ distribution of core, accessory, and unique genes.

### 2.7. Average Nucleotide Identity (ANI) Analysis

Average Nucleotide Identity (ANI) analysis was conducted to evaluate the pairwise genomic similarity among the 30 *X. oryzae* strains using the ANI calculator available online on JSpecies (https://jspecies.ribohost.com/jspeciesws/, accessed on 24 September 2024) [51]. Whole genome sequences in FASTA format were submitted for all-against-all ANI comparisons using the ANIb algorithm with default settings. The ANIb tool effectively assesses genome relatedness based on total nucleotide sequence similarity [52].

### 2.8. Genomic Alignment and Unique CDS Detection

To profile the unique genes across the genomes of the *X. oryzae* strains, the annotated genomes were aligned using the LASTZ alignment tool v.1.02.00 [53,54] on Geneious software v.9.1.8. The LASTZ alignment tool is relatively fast and effective in handling large genomic datasets. Unique CDS in each strain, absent in others, were identified. The intervals, protein IDs, and putative functions of the unique CDS in each strain were profiled and further analyzed to unravel their roles in the adaptability and pathogenicity of *X. oryzae*.

### 2.9. Phylogenetic Analysis

The phylogenetic trend among the *X. oryzae* strains was determined using the Codon Tree service hosted on the BV-BRC web server (https://www.bv-brc.org/app/PhylogeneticTree, accessed on 30 September 2024). Protein and nucleotide sequences from 100 randomly selected global Protein Families (PGFam) were aligned using MUSCLE [55] and MAFFT aligner [56], respectively, and then concatenated in PHYLIP format, from which the phylogenetic tree was inferred using RAxML (Randomized Axelerated Maximum Likelihood) [57]. The resulting Newick file was visualized using iTOL (Interactive Tree Of Life) v 6.9.1 [58] (https://itol.embl.de/, accessed on 30 September 2024).

### 2.10. Statistical Analysis

Tableau public (https://public.tableau.com/app, accessed on 4 October 2024) and GraphPad Prism (v.10) (GraphPad Software, Boston, MA, USA) were used for the statistical analysis and interactive visualization of the data.

## 3. Results

### 3.1. Genome Assembly Statistics

Supplementary Table S2 summarizes the genome assembly statistics of the 30 *X. oryzae* strains. The results show minor variations in assembly features of the *X. oryzae* strains. The genome lengths of the strains ranged from 4,948,537 bp to 5,118,699 bp, with a mean of 5,008,950 bp. In total, 40% of the strains had an above-average genome length. All the strains had a high GC content, ranging from 63.12% to 63.75%, and the number of contigs per strain ranged from one to seven. Only five strains—YNCX, NE-8, CIAT, IX-280, and CIX4508—had more than one contig; YNCX had the most contigs (seven), while the other strains all had one contig each, indicating a well-assembled genome. The average contig N50 value was 4,955,372 base pairs. Altogether, the dataset is adequate for further genomic analysis and comparisons within the species.

### 3.2. Functional Annotation

The genome annotation statistics for the 30 *X. oryzae* strains examined in this study are presented in Supplementary Table S2. The number of open reading frames (ORFs) ranged from 5017 to 5243; the number of annotated genes ranged from 3813 to 3976, with an average of 3886 per strain. Hypothetical proteins (proteins with no known functions) were also identified, varying from 1137 to 1340 among the strains. The non-coding regions were relatively stable across the strains, with only minor variations in the number of RNA genes, comprising both rRNA and tRNA, having 59 on average. The number of genes with the genus-specific Local Protein Families (PLfam) annotations varied from 4712 to 4923. More details of the annotation reports are presented in Supplementary Table S2.

### 3.3. Comprehensive Genome Analysis and Subsystem Categorization

**Figure 2:**
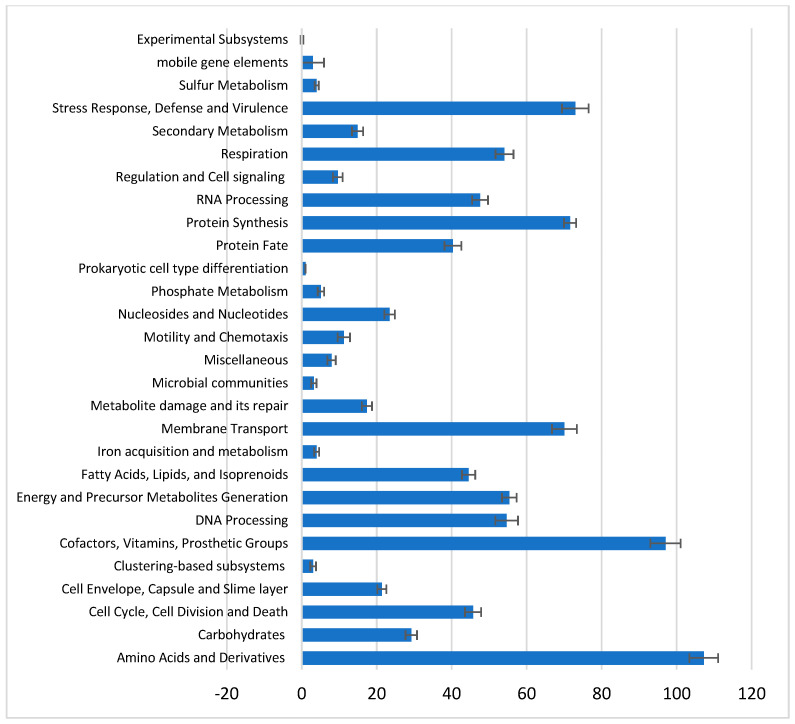
Functional subsystems categories and average number of corresponding genes in the genome of 30 *X. oryzae* strains. Bars represent mean values ± Standard deviation.

Comprehensive genome analysis reveals a high genetic variability across the metabolic and functional subsystems of the strains. Amino acid metabolism and carbohydrate metabolism genes varied between 99 and 116 and 25 and 32 genes, respectively, across the strains. The number of genes involved in the cell cycle, cell envelope structures, and energy metabolism were fairly stable, with an average of 46, 21, and 55 genes, respectively; similarly, genes involved in RNA metabolism and protein synthesis varied minimally. Wider variation was recorded in membrane transport genes and genes associated with virulence and responses to environmental stress; the same was observed in subsystems associated with cofactors, vitamins, and prosthetic groups. Iron metabolism genes were consistently low, and there was variation in genetic mobility, as shown by the absence of mobile genetic elements in several strains. The metabolic and functional diversity of *X. oryzae* strains provides insights into their pathogenicity, adaptation, and interactions with host plants. The overview of subsystems across the strains is presented in Figure 2.

### 3.4. ARG and VF Abundance and Distribution

**Figure 3:**
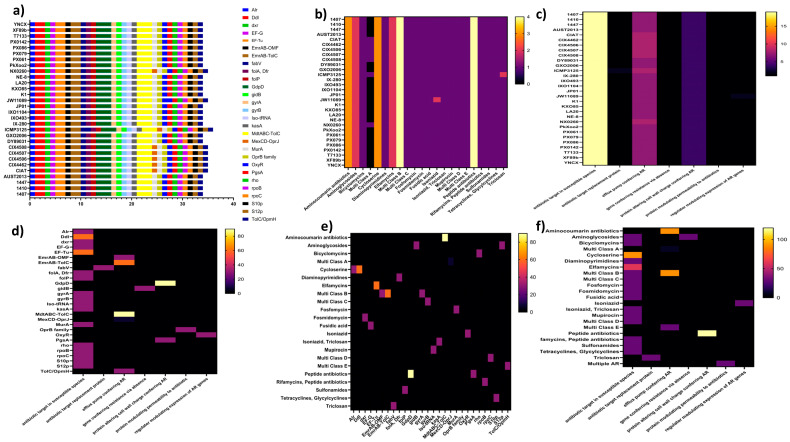
Abundance and functional mechanisms of AR genes in the *X. oryzae* strains. (**a**) Spatial distribution of AR genes within the genomes of *X. oryzae* strains. (**b**) *X. oryzae* strains’ resistance to antibiotics classes. (**c**) An overview of AR mechanisms across *X. oryzae* strains. (**d**) An overview of AR mechanisms exerted by AR genes identified in *X. oryzae* strains. (**e**) AR genes identified in *X. oryzae* genomes conferring resistance to antibiotic drug classes. (**f**) An overview of resistance mechanisms of *X. oryzae* strains to several antibiotic drug classes. **Antibiotics classes**: Multi-Class A: Cephalosporins, Penams, Tetracyclines, Phenicol antibiotics, Diaminopyrimidines, Aminocoumarin antibiotics, Aminoglycosides, Macrolides, Fluoroquinolones Quinolones Quinolines. Multi Class B: Fluoroquinolones Quinolones Quinolines. Multi-Class C: Fluoroquinolones Quinolones Quinolines, Aminocoumarin antibiotics. Multi Class D: Myxopyronins, Corallopyronins, Peptide antibiotics. Multi-Class E: Penams, Cephamycins, Cephalosporins, Monobactams, Tetracyclines, Rifamycins, Phenicol antibiotics, Aminocoumarin antibiotics, Fluoroquinolones Quinolones Quinolines, Triclosan, Macrolides, Glycylcyclines.

A total of 28 types of ARGs, both innate and acquired, were found in the resistome of the 30 *X. oryzae* strains (Figure 3a). The ARGs are associated with seven resistance mechanisms (Figure 3d), which include an antibiotic efflux pump, an antibiotic target in susceptible species, proteins that modify cell wall charge to confer AR, an antibiotic target replacement protein, resistance via absence, reduced permeability to antibiotics, and a regulator modulating the expression of AR genes. All strains have genes in each resistance mechanism; the most prevalent is “antibiotic target in susceptible species”, with an average of 19 member genes in each strain (Figure 3c). It is facilitated by several ARGs such as *Alr*, *Ddl*, *dxr*, *EF-G*, *EF-Tu*, *folA*, *Dfr*, *folP*, *gyrA*, *gyrB*, *Iso-tRNA*, *kasA*, *MurA*, *rho*, *rpoB*, *rpoC*, *S10p*, and *S12p*, conferring resistance to several antibiotic classes including aminoglycosides, bicyclomycins, cycloserine, diaminopyrimidines, elfamycins, fosfomycin, fosmidomycin, fusidic acid, isoniazid, triclosan, mupirocin, rifamycins, peptide antibiotics, sulfonamides, multi-class A, B, C, D, E, and tetracyclines/glycylcyclines. The most abundant ARG among the *X. oryzae* strains is *MdtABC-TolC* and *GdpD*, conferring resistance to aminocoumarin and peptide antibiotics, respectively. *EmrAB-OMF*, *EmrAB-TolC*, *MdtABC-TolC*, and *MexCD-OprJ* confer resistance via antibiotic efflux pumps; a resistance mechanism is also widespread among the strains, providing resistance to aminocoumarin antibiotics and multi-class antibiotics A, B, and E. Additionally, AR, by altering the cell wall charge, confers resistance to peptide antibiotics (Figure 3f). *X. oryzae* CIAT, CIX4462, CIX4506, CIX4507, CIX4508, ICMP3125, and NX0260 show potential resistance to all 22 antibiotic classes surveyed in this study, while other strains lacking *MexCD-OprJ* are likely susceptible to multiclass A antibiotics. These results depict the diversity of AR in *X. oryzae*; more details are presented in Figure 3a–f.

**Figure 4:**
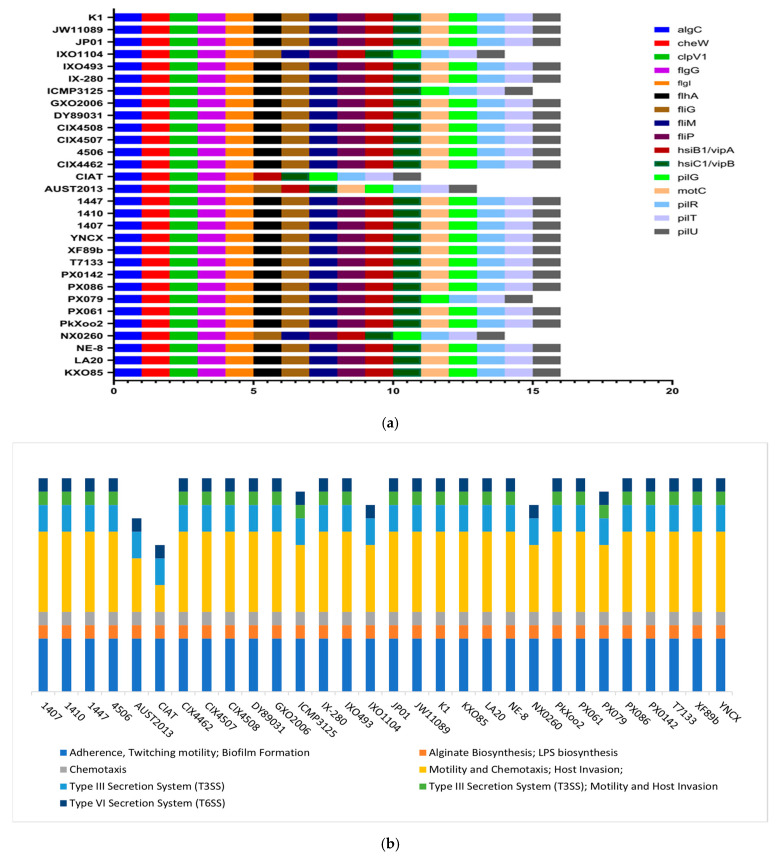
(**a**) Spatial distribution of virulence genes across the genomes of *X. oryzae* strains; (**b**) An overview of virulence mechanisms exerted by the virulence factors in each *X. oryzae* strain.

In total, 16 virulence factors were identified across the strains, contributing to the infestation of rice blight. These included *AlgC*, *CheW*, *ClpV1*, *FlgG*, *FlgI*, *HsiB1/VipA*, and *Hsi*. The *C1/vipB*, *pilG*, *pilR*, *pilT*, and *pilU* genes were present in all strains, while the others (*flhA*, *fliG*, *fliM*, *fliP*, *and motC*) showed a lower prevalence, ranging from 83 to 96% (Figure 4a). In total, 24 out of the 30 *X. oryzae* strains possessed all the identified genes. Six strains (NX0260, PX079, AUST2013, CIAT, ICMP3125, and IXO1104) had a lower number, with 11 to 15 genes present. These genes participate in several processes fostering the strains’ pathogenicity, which include adherence, twitching motility, biofilm formation, alginate biosynthesis, LPS biosynthesis, chemotaxis, and Type III and VI secretion systems (T3SS, T6SS). All strains except NX0260, AUST2013, CIAT, and IXO1104 exhibited all virulence mechanisms identified in this study (Figure 4b). The virulence genes identified and their gene products are presented in Table 1.

### 3.5. Distribution and Diversity of Transposable Elements Among X. oryzae Strains

**Figure 5:**
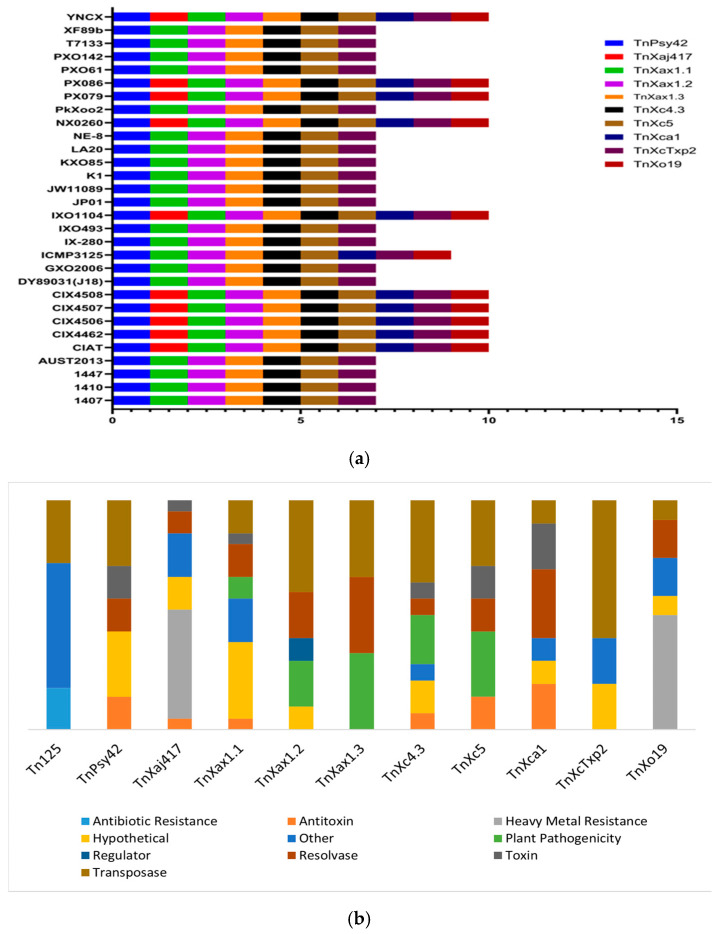
(**a**) Spatial distribution of transposons across *X. oryzae* strains’ genomes; (**b**) Functional annotation of gene contents of transposons identified across the *X. oryzae* strains.

Several transposable elements were identified and spread among the strains of *X. oryzae* assessed in this study. All 30 strains harbored the transposons *Tn125*, *TnPsy42*, *TnXax1.1*, *TnXax1.2*, *TnXax1.3*, *TnXc4.3*, *TnXc5*, and *TnXcTxp2.* However, the distribution of *TnXaj417*, *TnXca1*, and *TnXo19* varied across the strains, with *TnXaj417* present in only ten strains, while *TnXca1* and *TnXo19* were detected in 11 strains each (Figure 5a). The strains with the highest diversity of transposons were CIAT, CIX4462, CIX4506, CIX4507, CIX4508, ICMP3125, IXO1104, NX0260, PX079, PX086, and YNCX; all except ICMP3125 had 11 elements. A more limited transposon profile was seen in strains such as 1407, 1410, 1447, AUST2013, DY89031, GXO2006, IX-280, IXO493, JP01, JW11089, K1, KXO85, LA20, NE-8, PkXoo2, PXO61, PXO142, T7133, and XF89b, which did not include *TnXaj417*, *TnXca1*, and *TnXo19*.

A comprehensive analysis of the identified transposon genes contents sheds light on their functional roles and impact on host strains’ adaptability (Figure 5b). *TnXo19*, a transposon largely associated with heavy metal resistance, carried genes such as *czcC*, *czcB*, and *czcA*, which confer resistance to cobalt, zinc, and cadmium. Other important genes include *hupE_ureJ*, associated with membrane protein function; *yfiS*, an MFS transport protein; *secC*, a zinc chelator; and putative proteins like WP_150411558.1.

In *TnXca1*, transposase genes like *tnpA* were found with toxin–antitoxin systems like the *zeta*, *epsilon*, *PIN*, and *abrB* genes. *TnXcTxp2* had plasmid transfer-related genes such as *B7L66_25000 and mobAL.* The transposons *TnXax1.1*, *TnXax1.2*, and *TnXax1.3* had transposase elements (*tnp*, *tnpA*, *and orfAB*), as well as genes associated with plant pathogenicity such as *mltB*, *avr*, and *xopC*. Likewise, a virulent gene, *‘SecC’*, was discovered in *TnXc4.3*, *TnXc5*, and *TnXax1.3*, promoting pathogenicity. *Tn125* contains certain genes (*MRP(MBL*) and *bla NDM-2*) with known antibiotic resistance roles, enhancing host resistance to several antibiotic classes. More details about the transposons’ gene contents are presented in Figure 5b. The results reveal transposons’ silent impact on host strains’ adaptability and virulence.

The analysis shows little variation in the distribution of insertion sequences (IS) among the strains examined. Specifically, transposons such as *IS1051*, *IS1112*, *IS1112a*, *IS1112b*, *IS1113*, *IS1114*, *IS1389*, *IS1389-B*, *IS1403*, *IS1404*, *IS1478*, *IS1479*, *IS1481A*, *IS1595*, *IS1646*, *ISCARN25*, *ISPa104*, *ISPsy42*, *ISXac1*, *ISXac3*, *ISXaca1*, *ISXc4*, *ISXc6*, *ISXca4*, *ISXca5*, *ISXfu1*, *ISXfu2*, *ISXo1*, *ISXo2*, *ISXo3*, *ISXo4*, *ISXo5*, *ISXo7*, *ISXo9*, *ISXo14*, *ISXo15*, *ISXo16*, *ISXo17*, *ISXo18*, *ISXoo4*, *ISXoo5*, *ISXoo7*, *ISXoo13*, and *ISXoo14* were present across all strains, suggesting they are conserved within the species.

**Figure 6:**
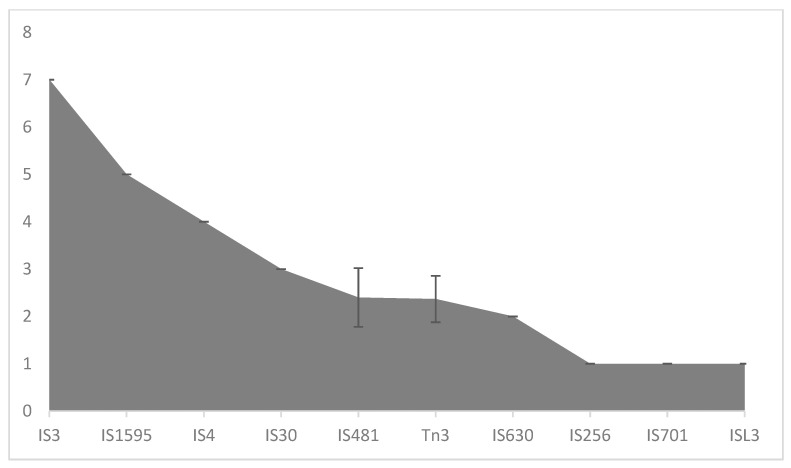
Distribution of insertion sequence families in 30 *X. oryzae* strains. Peaks represent the mean value of individual families across the 30 *X. oryzae* strains, with the standard deviation from the mean as error bars.

Specific IS have staggered distribution among the strains. The *ISVei2* transposon was absent in the *X. oryzae* strains CIAT and NX0260; likewise, *ISVei3* was not found in AUST2013, JP01, PX086, GXO2006, JW11089 and 11 other strains. The distribution of the IS across the genomes, based on IS families, is presented in Figure 6.

### 3.6. Unique Coding Sequences Among the Strains

**Figure 7:**
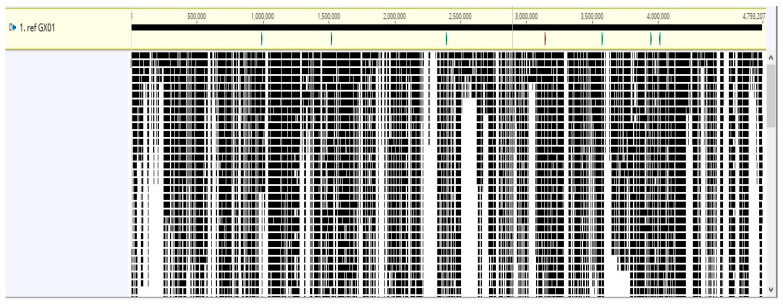
Whole genome alignment of the *X. oryzae* strains reveals genetic variation and unique coding regions.

**Figure 8:**
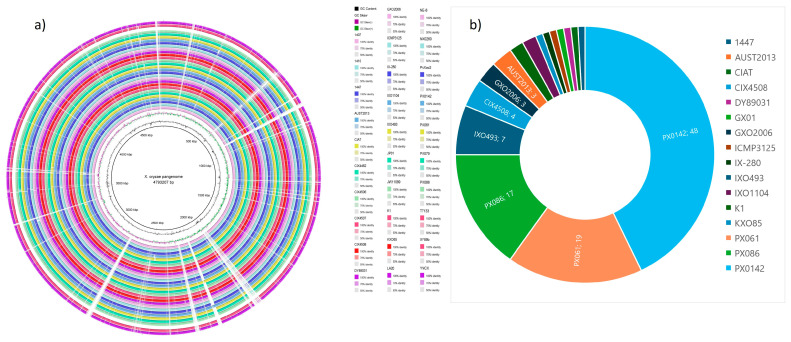
An overview of the (**a**) pangenome and (**b**) unique coding sequences across the *X. oryzae* strains.

A total of 112 unique genes were discovered across the strains. Strains collected in the Philippines, namely, PX0142, PX061, and PX086, had the most unique genes (48, 19, and 17, respectively), with diverse metabolic, virulence, and regulatory roles. The *X. oryzae* strain 1447 hosted a unique gene that encodes Homoserine O-acetyltransferase (WP_149621534.1) with a speculated function in the metabolism of amino acids. A DUF1294 domain-containing protein, an IS5 family transposase, and a SymE family type I addiction module toxin (WP_149621668.1) were found in strain AUST2013 and could enhance genetic mobility and the stress response. A well-known virulence factor *avrBs3* gene (WP_149621514.1), an effector protein that distorts the host plant cellular processes, is featured in strains CIAT, K1, and CIX4508; in addition, strain CIX4508 also hosted multiple transposases from the IS30 and IS5 families. The *avrBs2* gene is also an effector protein that promotes infection by degrading host plant proteins featured in strain PX061. Figure 7 and Figure 8a give an overview of intraspecific variations in the genome of the *X. oryzae* strains.

Among the other discoveries are strains GX01 and DY89031, the former of which contains an *IS30* family transposase (WP_014502919.1) and the latter contains an IS30-like element of the IS1112a family also found in strains CIX4508, K1, and PX086. Transposable elements such as *IS5*-like transposases (WP_011258802.1, WP_149621975.1) and an *IS5* family transposase featured in strain GXO2006 enhance genome plasticity. Several novel genes, including *tssI* (WP_263433141.1), an AAA family ATPase (WP_149621553.1), transposases, the T6SS *Vgr* family protein, and several phage late control D family proteins, are found in strain IXO493. These proteins could participate in secretion systems and phage interaction. The presence of a two-component regulator propeller domain-containing protein (WP_048484041.1) is exclusive to strain KXO85. More details are presented in Figure 8.

### 3.7. Pangenome Analysis and Phylogenetic Relationship

**Figure 9:**
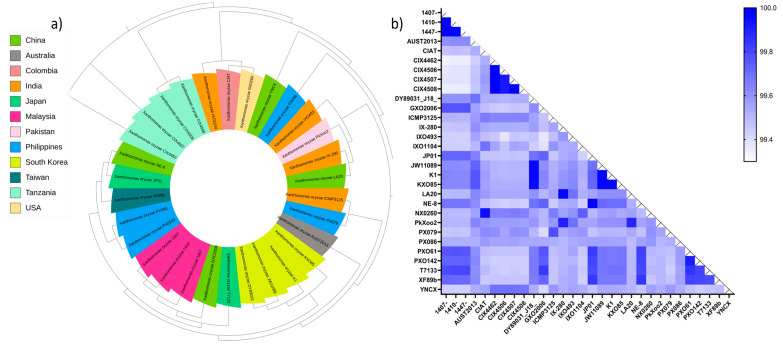
(**a**) Phylogenetic relationship and (**b**) ANI between *X. oryzae* strains collected from different geographical regions across five continents. The heatmap displays pairwise ANI values between the *X. oryzae* strains. Color intensity corresponds to ANI percentage, with darker colors indicating higher similarity.

Analyses of the pangenome (Figure 8a), ANI, and phylogenetic relationships (Figure 9) reveal genetic diversity and intraspecific evolution among the *X. oryzae* population. Although little variability exists among the Tanzanian strains, namely, CIX4462, CIX4506, CIX4507, and CIX4508, having above 99.8 % nucleotide identity forming the most basal clade with a bootstrap value of 100; likewise, KXO85, K1, JW11089, and DY89031 from South Korea (ANI > 99.6%;bootstrap = 98) forming unique subclades with the Malaysian strains, *X. oryzae* strains collected from India, the Philippines, China, and Japan show a high level of evolutionary divergence with member strains across several clades and sub-clades. The phylogenetic profile of the *X. oryzae* strains and an overview of their average nucleotide identity are presented in Figure 9.

## 4. Discussion

Bacterial blight disease, caused by the bacterium *X. oryzae* pv. *Oryzae*, has become a global phenomenon, with several outbreaks across rice-growing regions, causing significant yield losses of up to 50% [24]. As the world strives to achieve food security in the coming decades, the heightened spread and outbreaks of bacterial blight disease in rice plantations must be addressed. The adequate profiling of the virulence factors, AR genes in *X. oryzae*, and their mechanisms of interaction with the host plant defense system is essential for developing effective control strategies [10,31].

The genome subsystem categorization of the strains assessed in this study shows wide variation in the genes associated with membrane transport, virulence, and responses to environmental stress (Figure 2). This reflects a differential pathogenicity and resilience among the strains of *X. oryzae*, as reported earlier [10]. The abundance of AR genes in *X. oryzae*’s genome has a major impact on its ability to adapt and establish infection in its host plant. In total, 29 unique AR genes were identified across the strains (Figure 3a). Previous reports [59,60] and recognized databases [42,61] have confirmed the AR roles of these genes in host organisms. They increase the pathogen’s capacity to survive various antibiotic treatments, such as fluoroquinolones, quinolones, peptide antibiotics, tetracyclines, and glycylcyclines. The high frequency of these genes in *X. oryzae* populations makes treatment and control challenging [31].

Among the six AR mechanisms identified in the study population, antibiotic target modification is the most common (Figure 3c), with several participating genes in each strain, confirming earlier reports [32]. This mechanism, coupled with antibiotic target replacement proteins in all strains, modifies or completely replaces the antibiotic target site [62], inducing resistance to multiple antibiotic classes often used by rice farmers to control the pathogen. Efflux pumps and cell wall charge alteration–AR mechanisms are also prevalent among the strains, actively ejecting antibiotics out of the bacterial cell [63,64] or modifying its cell wall to reduce antibiotic permeability [32]. There are also instances of antibiotic-susceptible gene deletions conferring resistance on the strains.

Multiple AR systems in pathogens represent a significant global concern, complicating disease management efforts. Prior research has indicated that numerous antibiotic classes are becoming ineffective and redundant in controlling *X. oryzae* [65,66]. The strains demonstrate a degree of uniformity in their response to various antibiotic classes (Figure 3b). The results show that each of the strains possesses one or more genes that potentially confer resistance to a multitude of antibiotic classes, including aminocoumarin antibiotics, aminoglycosides, bicyclomycins, peptide antibiotics, cycloserine, diaminopyrimidines, elfamycins, fosmidomycin, fusidic acid, isoniazid, and triclosan, among others (Figure 3e). The resistance observed in this study may be widespread within the species. The high degree of AR observed in *X. oryzae* raises a serious concern about the potential leakage of AR genes into the environment.

A distinctive resistome profile was detected in CIAT and the Tanzanian strains, having a unique AR gene, ‘*MexCD-OprJ*’ a multidrug efflux system. This could have emerged from selective pressure, especially the use of multiple antibiotics in bacterial rice blight management and other region-specific adaptation demands [67], which influences the evolution of AR in *X. oryzae* populations. This gene confers additional resistance to antibiotics in ’multi-class A’ comprising the cephalosporins, penams, tetracyclines, phenicols, diaminopyrimidines, aminocoumarins, aminoglycosides, macrolides, fluoroquinolones, and quinolines antibiotic classes. Therefore, it is highly necessary to regulate the use of antibiotic drugs for *X. oryzae* rice blight disease management in these regions, as new AR traits could still emerge [68]. Attention should be shifted to fully adapting resistant rice varieties and bio-control strategies. Despite reports of the uneven and insufficient circulation of improved planting material in African sub-hubs [69,70], adequate intervention by governmental and non-governmental bodies can achieve the desired outcome. Concerted scientific and agronomic attention is also required to prevent outbreaks by these strains and avoid huge economic losses to rice farmers and the further spread of AR genes in the region [71]. The high frequency of AR genes within *X. oryzae* populations complicates control efforts; these genes need continuous monitoring and profiling for effective management [31].

Virulence genes in *X. oryzae* play key roles in host plant invasion and rice plantation infestation. Their distribution and abundance should be adequately profiled to understand the pathogenesis of bacterial blight in rice [72,73]. We identified many virulence genes in the study population (Figure 4a), supporting earlier findings [72]. Tall et al. [74] also reported varying virulence profiles among some Senegalese races of *X. oryzae*, depicting vast genetic diversity within its populations. There is a continual evolution of virulence factors in this species as they strive to overcome host barriers [72]; Quibod et al. [14] made a similar observation among the Philippine population of the pathogen, evolving different virulence mechanisms to circumvent the *Xa4* gene in resistant rice varieties, confirming the multidimensional virulence potential of *X. oryzae.* Our stringent blast condition identified 16 genes contributing to its virulence, all present in 80% of the strains. The type VI secretion system (*hsiB1/vipA* and *hsiC1/vipB*), adhesion and twitching motility (*pilG*, *pilR*, *pilT*, and *pilU*), and motility and chemotaxis (*flgG and flgI*) are among the genes that were conserved and present in all the strains. These genes have been identified as key players in the pathogenesis of *X. oryzae* [75,76].

Virulence genes such as *algC*, *cheW*, and the Type III and VI Secretion System component genes (TSS) present across the strains establish their importance in the pathogen’s virulence regarding host plants. The *cheW* gene and genes of the Type IV pilus (T4P) system, which are also abundant in the study strains, aid the pathogen’s motility towards targeted host tissues to establish infections [77]. The *algC* gene is equally important, functioning alongside the motility genes. It participates in alginate biosynthesis in microbes and is also involved in biofilm formation [78], protecting the pathogens from host plant defense systems and aiding invasion. These genes are present across the strains, confirming earlier reports of their importance in *X. oryzae* pathogenicity [11]. The results also show the pathogen reliance on Type III secretion systems for delivering effector proteins into plant cells, with an abundance of genes associated with the secretion system, such as *flhA*, *hsiB1/vipA*, and *hsiC1/vipB*. Effector proteins play down the host plant’s immune system, facilitating infection [79].

Transposons and insertion sequence (IS) elements, abundant in the *X. oryzae* genome, have significantly shaped its evolution [80]. These genetic elements cause deletions, rearrangements, and other genomic changes in addition to facilitating lateral gene transfer, making the strains flexible for easy adaptation. These alterations can occur in the acquisition, loss, or alteration of gene content [80]. Our study identified several transposons in the strains’ genome, bearing genes contributing to traits such as AR, pathogenicity, heavy metal resistance, antitoxin, and gene expression regulation (Figure 5b). The transposons *TnXax1.1*, *TnXax1.2*, *TnXax1.3*, *TnXc4.3*, and *TnXc5* present in all strains assessed carry virulence genes such as *secC* and *xopC*—type III secretion system effector proteins; *mltB*—maintaining cell wall integrity to withstand the host plant defense system [81]; and *avr*—encoding an effector protein [82,83]. Similarly, the transposons *Tn125*, *TnXaj417*, and *TnXo19* bore genes conferring antibiotic and heavy metal resistance on host strains. The impact of transposons on *X. oryzae*’s pathogenicity and adaptability is evident. These mobile elements facilitate the horizontal transfer of genes among the population and neighboring species, promoting AR resistance and virulence [84].

The detection of unique coding regions among the *X. oryzae* strains affirms earlier findings of environment-oriented, intra-specific divergent evolution among the species population [24]. Sixteen strains have unique coding sites, the highest in strain PX0142 (48) (Figure 8b). These strains have evolved specific mechanisms to adapt to their environment, resist antibiotics, and interact with their hosts. The unique genes influence diverse functions, including AR, pathogenicity, stress responses, and DNA repair. The clustering of strains from Africa and United strains in close proximity, as observed in this study, has been reported earlier [26], suggesting a common ancestor. Strains from China, India, the Philippines, and Japan show considerable genetic divergence, further depicting intraspecific variability among the *X. oryzae* population. This reflects the genetic adaptability of these strains to environmental dictates and host plant defense systems; notably, these regions are among those most hit by *X. oryzae* infestation [85]. This rapid evolution and genetic variability necessitate targeted control measures; one-for-all management strategies will not be effective. We also observed phylogenetic clusters comprising strains from different regions, suggesting a common ancestor. This calls for a more stringent inter-border quarantine control system to avoid the spread of this virulent pathogen across borders.

## 5. Conclusions

Antibiotic resistance poses a major threat to agricultural productivity and *X. oryzae* management. The growing surge of AR in *X. oryzae*, coupled with its genomic flexibility and adaptability to overcome host defense systems, promotes its spread and will further complicate control efforts in the coming years. More attention is needed to curb the pathogen. There is an urgent need to shift from a drug-based management approach to adopting resistant rice varieties and biocontrol systems globally. This will mitigate the ongoing AR gene emergence in *X. oryzae* and increase rice farmers’ production capacity in the highlighted countries. The findings from this study elucidate the pathogen’s pathogenicity and adaptability dynamics; the credible information provided can be integrated into breeding strategies to develop novel management strategies, such as rice varieties with broad-spectrum resistance.

## Figures and Tables

**Table 1:** Virulence genes identified in *X. oryzae* strains, molecular mechanisms, and gene products.

***Genes***	**Classification**	**Product**
*algC*	Alginate Biosynthesis; LPS biosynthesis	phosphomannomutase AlgC [Alginate biosynthesis (CVF522)]
*cheW*	Chemotaxis	chemotaxis protein CheW [Flagella (VF0430)]
*clpV1*	Type VI Secretion System (T6SS)	type VI secretion system AAA+ family ATPase [HSI-I (VF0334)]
*flgG*	Motility and Chemotaxis; Host Invasion;	flagellar basal-body rod protein FlgG [Flagella (VF0273)]
*flgI*	Motility and Chemotaxis; Host Invasion;	flagellar P-ring protein precursor FlgI [Flagella (VF0273)]
*flhA*	Type III Secretion System (T3SS); Motility and Host Invasion	flagellar biosynthesis protein FlhA [Flagella (VF0273)]
*fliG*	Motility and Chemotaxis; Host Invasion;	flagellar motor switch protein G [Flagella (VF0273)]
*fliM*	Motility and Chemotaxis; Host Invasion;	flagellar motor switch protein FliM [Flagella (VF0273)]
*fliP*	Motility and Chemotaxis; Host Invasion;	flagellar biosynthetic protein FliP [Flagella (VF0273)]
*hsiB1/vipA*	Type III Secretion System (T3SS)	(hsiB1/vipA) type VI secretion system tubule-forming protein VipA [HSI-I (VF0334)]
*hsiC1/vipB*	Type III Secretion System (T3SS)	(hsiC1/vipB) type VI secretion system tubule-forming protein VipB [HSI-I (VF0334)]
*motC*	Motility and Chemotaxis; Host Invasion;	flagellar motor protein [Deoxyhexose linking sugar 209 Da capping structure (AI138)]
*pilG*	Adherence, Twitching motility; Biofilm Formation	twitching motility protein PilG [Type IV pili (VF0082)]
*pilR*	Adherence, Twitching motility; Biofilm Formation	two-component response regulator PilR [Type IV pili (VF0082)]
*pilT*	Adherence, Twitching motility; Biofilm Formation	twitching motility protein PilT [Type IV pili (VF0082)]
*pilU*	Adherence, Twitching motility; Biofilm Formation	twitching motility protein PilU [Type IV pili (VF0082)]

## Data Availability

The original contributions presented in the study are included in the article; further inquiries can be directed to the corresponding author.

## Supplementary Materials

The following supporting information can be downloaded at https://www.mdpi.com/article/10.3390/life14121690/s1, Table S1: Geographical source, collection, and submission information of the *X. oryzae* strains; Table S2: Genome assembly statistics and annotation reports of 30 *X. oryzae* strains.
