# Supplementary material for: Pathogenomic Insights into Xanthomonas oryzae pv. oryzae’s Resistome, Virulome, and Diversity for Improved Rice Blight Management
Source: Life (Basel). 2024 Dec 20;14(12):1690. doi: 10.3390/life14121690 (PMC11678079; doi:10.3390/life14121690)
Supplement: Supplementary file 1 [file life-14-01690-s001.zip › life-3347100-supplementary.pdf]

Supplementary Table S1: Geographical source, collection and submission information of the *X. oryzae* strains

Sample ID	Bioproject	Biosample	SRA	Genome length	Geo Region	Isolation source	Submitter	Year	Sequencing platform	Assembly	Size
AUST2013	PRJNA497605	SAMN10261811	SRS4581623	4,958,184	Australia		University of Florida, Jose Huguet-Tapia	2019	PacBio	GCA_004355785.1	5 Mb
YNCX	PRJNA861373	SAMN29911672		4,978,095	China, Yunnan Plateau	Leaves of the Japonica rice variety	Yunnan Academy of Agricultural Sciences	2022	PacBio RSII; Illumina HiSeq	GCF_024499285.1	5.1 Mb
LA20	PRJNA913657	SAMN32303221		4,960,087	China: Anhui	isolated from rice leaf	China National Rice Research Institute	2022	PacBio Sequel; Illumina NovaSeq	GCF_027257035.1	5 Mb
NE-8	PRJNA919198	SAMN32623252		4,986,881	China: Mudanjiang		Chinese Academy of Agricultural Sciences	2023	PacBio	GCF_029542325.1	5 Mb
GXO2006	PRJNA775854	SAMN22630272	SRS10777363	4,966,657	China: Guangxi	infected rice leaf	Guangxi University	2021	PacBio Sequel	GCF_020790135.1	5 Mb
CIAT	PRJNA497605	SAMN10261812	SRS4581630	5,035,285	Colombia		University of Florida	2019	PacBio	GCA_004355865.1	5.1 Mb
ICMP3125	PRJNA485465	SAMN09791860		4,990,672	India	leaf blight and wilt	Martin Luther University Halle-Wittenberg	2019	PacBio RSII	GCA_004136375.1	5 Mb
IXO1104	PRJNA269488	SAMN03252587		5,093,432	India		CSIR-Institute of Microbial Technology	2022	Oxford Nanopore MiniION; Illumina MiSeq	GCA_001929245.2	5.0 Mb
IXO493	PRJNA269466	SAMN03252574		5,088,542	India		CSIR-Institute of Microbial Technology	2022	Oxford Nanopore MiniION; Illumina MiSeq	GCA_001928825.2	5.1 Mb
IX-280	PRJNA330733	SAMN05717683	SRS2477181	4,963,594	India: Andhra Pradesh, Tanuku	rice leaves	ICAR-National Research Centre on Plant Biotechnology	2018	PacBio	GCF_003427055.1	5 Mb
T7133	PRJNA715045	SAMN18324990		4,978,475	Japan		Kindai University	2021	PacBio RSII	GCF_017577225.1	5 Mb
JP01	PRJNA350904	SAMN05953100	SRS1785463	4,948,537	Japan	rice leaves	Huazhong Agricultural University	2018	PacBio	GCF_003382855.1	4.9 Mb
1447	PRJNA743781	SAMN20060269		4,954,304	Malaysia: Keda		MARDI	2022	Illumina	GCF_023277425.1	5 Mb
1410	PRJNA743781	SAMN20060268		4,954,304	Malaysia: Perlis		MARDI	2022	Illumina	GCF_023277405.1	5 Mb
1407	PRJNA743781	SAMN20060267		4,954,304	Malaysia: Selangor		MARDI	2022	Illumina	GCF_023277385.1	5 Mb

PkXoo2	PRJNA861287	SAMN37852903		4,941,434	Pakistan: Punjab		Plant-Microbe Interaction lab, NIBGE	2024	Oxford Nanopore	GCF_039555255.1	4.9 Mb
PX079	PRJNA350904	SAMN05953163	SRS1785613	5,026,592	Philippines	rice leaves	Huazhong Agricultural University	2018	PacBio	GCA_003382895.1	5 Mb
PX086	PRJNA350904	SAMN05953164	SRS1785579	5,015,823	Philippines	rice leaves	Huazhong Agricultural University	2018	Illumina HiSeq	GCF_003382915.1	5 Mb
PXO61	PRJNA381940	SAMN06695275	SRS2127295	4,999,360	Philippines		International Rice Research Institute	2018	Illumina	GCF_003122405.1	5 Mb
PXO142	PRJNA485467	SAMN09791863		4,982,118	Philippines	leaf blight and wilt	Martin Luther University Halle-Wittenberg	2019	PacBio RSII	GCF_004136395.1	5 Mb
K1	PRJNA608233	SAMN14167900		4,981,423	South Korea	infected leaf tissue	National Institute of Crop Science	2020	Illumina	GCF_011045315.1	5 Mb
DY89031(J18)	PRJNA675962	SAMN16744833		4,979,456	South Korea		CAS Center for Excellence in Molecular Plant Sciences	2021	PacBio RSII	GCF_018288775.1	5 Mb
JW11089	PRJNA497605	SAMN10261813	SRS4581629	5,010,511	South Korea		University of Florida	2019	PacBio	GCF_004355765.2	5 Mb
KXO85	PRJNA497605	SAMN10261815	SRS4581624	4,975,259	South Korea		University of Florida	2019	PacBio	GCF_004355845.1	5 Mb
XF89b	PRJNA284661	SAMN03729481	SRS2863163	4,966,744	Taiwan: Taichung		Academia Sinica	2017	Illumina; Sanger dideoxy sequencing	GCF_002023005.1	5 Mb
CIX4462	PRJNA860955	SAMN29872137		5,150,745	Tanzania: Dakawa	Leaf	Institut de Recherche pour le Developpement	2023	Oxford Nanopore + Illumina NextSeq	GCA_029204285.1	5.2 Mb
CIX4507	PRJNA860955	SAMN33312895		5,134,670	Tanzania: Lukenge		Institut de Recherche pour le Developpement	2023	Oxford Nanopore + Illumina NextSeq	GCA_029224685.1	5.1 Mb
CIX4508	PRJNA860955	SAMN35102727		5.1 Mb	Tanzania: Lukenge		Institut de Recherche pour le Developpement	2023	Oxford Nanopore + Illumina NextSeq	GCA_030056695.1	5.1 Mb
CIX4506	PRJNA860955	SAMN29872138		5,109,772	Tanzania: Mvomero		Institut de Recherche pour le Developpement	2023	Oxford Nanopore + Illumina NextSeq	GCA_029204265.1	5.11
NX0260	PRJNA497605	SAMN10261817		5,050,391	USA		University of Florida	2019	PacBio	GCA_004355825.1	5.1 Mb

Supplementary Table S2: Genome assembly statistics and annotation reports of 30 *X. oryzae* strains

Sample ID	Plasmids	Genome Length (bp)	GC Contents (%)	Open Reading Frame	Annotated Genes	Hypothetical Proteins	RNA Genes	CRISPR Repeats	Genes with PLfam	Genes with PGfam	Contigs N50	Contigs
AUST2013	0	4,958,184	63.71	5,017	3,880	1,137	60	74	4,733	4,837	4,958,184	1
YNCX	0	5118699	63.12387	5235	3895	1340	64	108	4791		4978095	7
LA20	0	4,958,184	63.71	5,017	3,880	1,137	60	74	4,733	4,837	4,958,184	1
NE-8	0	5,004,028	63.69	5,152	3,893	1,259	59	47	4,858	4,978	4,986,881	2
GXO2006	0	4,966,657	63.71	5,109	3,848	1,261	59	79	4,798	4,921	4,966,657	1
CIAT	0	5,104,724	63.63	5,180	3,898	1,282	59	33	4,875	5010	5,035,285	2
ICMP3125	0	4,990,672	63.69	5,067	3,875	1,192	59	69	4,782	4,899	4,990,672	1
IXO1104	0	5,093,432	63.68	5,165	3,913	1,252	60	110	4,888	5,007	5,093,432	1
IXO493	0	5,088,542	63.64	5,243	3,976	1,267	59	65	4,920	5,053	5,088,542	1
IX-280	0	5,006,569	63.67	5,147	3,901	1,246	59	90	4,857	4969	4,963,594	2
T7133	0	4978475	63.68	5120	3854	1266	60	68	4808	4936	4978475	1
JP01	0	4,948,537	63.69	5,073	3,847	1,226	59	49	4,780	4,898	4,948,537	1
1447	0	4,954,304	63.75	5,020	3,813	1,207	58	58	4,712	4,827	4,954,304	1
1410	0	4,954,304	63.75	5,029	3,821	1,208	58	58	4,714	4829	4,954,304	1
1407	0	4,954,304	63.75	5,046	3,814	1,232	58	58	4,731	4,841	4,954,304	1
PkXoo2	0	4,941,434	63.69	5,065	3,856	1,209	59	59	4,769	4,878	4,941,434	1
PX079	0	5,026,592	63.65	5,097	3,877	1,220	59	76	4,815	4,942	5,026,592	1
PX086	0	5,015,823	63.72	5,048	3,856	1,192	60	88	4,790	4,901	5,015,823	1
PXO61	0	4,999,360	63.68	5,144	3,904	1,240	60	84	4,833	4,955	4,999,360	1
PXO142	0	4,982,118	63.69	5,103	3,917	1,186	60	99	4,789	4,911	4,982,118	1
K1	0	4,981,423	63.7	5,114	3,874	1,240	60	74	4,809	4,929	4,981,423	1
DY89031(J18)	0	4,979,456	63.69	5,107	3,870	1,237	59	73	4,811	4,929	4,979,456	1
JW11089	0	5,010,511	63.69	5,116	3,886	1,230	59	76	4,819	4,937	5,010,511	1

KXO85	0	4,975,259	63.69	5,111	3,862	1,249	60	60	4,803	4,923	4,975,259	1
XF89b	0	4,966,744	63.7	5,086	3,854	1,232	59	83	4,795	4,914	4,966,744	1
CIX4462	0	5,150,745	63.6	5,210	3,964	1,246	59	68	4,915	5,036	5,150,745	1
CIX4507	0	5,134,670	63.61	5,214	3,946	1,268	59	68	4,923	5,041	5,134,670	1
CIX4508	0	5,131,688	63.59	5,193	3,951	1,242	59	68	4,900	5,018	4,321,040	2
CIX4506	0	5,109,772	63.6	5,157	3,932	1,225	59	68	4,862	4,978	5,109,772	1
NX0260	0	5,050,391	63.67	5,138	3,924	1,214	59	33	4,846	4,972	5,050,391	1
